# Supplementary material for: Analysis of PPARγ Signaling Activity in Psoriasis
Source: Int J Mol Sci. 2021 Aug 10;22(16):8603. doi: 10.3390/ijms22168603 (PMC8395241; doi:10.3390/ijms22168603)
Supplement: Supplementary file 1 [file ijms-22-08603-s001.zip › Supplemental materials_Analysis of PPARg signaling activity in psoriasis/Pathway models/Models images and html files/PPARG expressed in cells assotiated with psoriasis/225946.html]

neutrophil


# CellType neutrophil

|  |  |
| --- | --- |
| URN | urn:agi-ncimcelltype:C0027950 |
| Total Entities | 69 |
| Connectivity | 10357 |
| Name | neutrophil |

---

|  |  |
| --- | --- |
| ChildConcepts | neutrophil function |
|  | neutrophil adhesion |
|  | neutrophil migration |
|  | neutrophil aggregation |
|  | neutrophil extravasation |
|  | neutrophil homeostasis |
|  | neutrophil apoptosis |
|  | neutrophil differentiation |
|  | neutrophil chemotaxis |
|  | neutrophil sequestration |
|  | neutrophil infiltration |
|  | neutrophil rolling |
|  | neutrophil survival |
|  | neutrophil transendothelial migration |
|  | neutrophil proliferation |
|  | neutrophil formation |
|  | neutrophil phagocyte activity |
|  | neutrophil homing |
|  | neutrophil invasion |
|  | neutrophil interaction |
|  | neutrophil death |
|  | neutrophil polarity |
|  | neutrophil autophagy |
|  | neutrophil phenotype |
|  | neutrophil phagocytosis |
|  | neutrophil transepithelial migration |
|  | neutrophil development |
|  | neutrophil diapedesis |
|  | neutrophil priming |
|  | neutrophil population |
|  | neutrophil selection |
|  | neutrophil motility |
|  | neutrophil growth |
|  | neutrophil lifespan |
|  | neutrophil agglutination |
|  | neutrophil contact |
|  | neutrophil motion |
|  | neutrophil fate |
|  | neutrophil renewal |
|  | neutrophil ratio |
|  | neutrophil mediated cytotoxicity |
|  | neutrophil senescence |
|  | neutrophil size |
|  | neutrophil localization |
|  | neutrophil macrophage differentiation |
|  | neutrophil fate determination |
|  | neutrophil volume |
|  | neutrophil damage |
|  | neutrophil tethering |
|  | neutrophil spreading |
|  | neutrophil destruction |
|  | neutrophil fate specification |
|  | neutrophil repertoire |
|  | neutrophil transdifferentiation |
|  | neutrophil cytotoxicity |
|  | neutrophil recognition |
|  | neutrophil communication |
|  | neutrophil swelling |
|  | neutrophil disruption |
|  | neutrophil regeneration |
|  | neutrophil stress |
|  | neutrophil distribution |
|  | neutrophil lymphocyte proliferation |
|  | neutrophil compartmentalization |
|  | neutrophil elongation |
|  | neutrophil fate commitment |
|  | neutrophil granulocyte differentiation |
|  | neutrophil developmental process |
|  | neutrophil architecture |

---

|  |  |
| --- | --- |
| Pathway | Keep out Signal: LTF Inhibits Neutrophil Recruitment and Inflammation |
|  | Myoblast -> Neutrophil Surface Expression Markers |
|  | PPARG expressed in cells assotiated with psoriasis |
|  | Neighbors of neutrophil |
|  | 2\_3\_Eosinophilia and neutrophilia in asthma: Neutrophil chemotaxis and activation |
|  | 1\_SERPINA1-associated pulmonary emphysema |

---

|  |  |
| --- | --- |
| MedScan ID | 10000412 |

---

|  |  |
| --- | --- |
| Alias | polymorphonuclear granulocytes |
|  | neutrophil leukocytes |
|  | polymorphous leukocyte |
|  | neutrophil granulocyte |
|  | neutrophilic granulocyte |
|  | polymorphonuclear leukocyte |
|  | neutrocyte |
|  | polymorphonuclear granulocyte |
|  | pmn leukocytes |
|  | polymorphonuclear leukocytes |
|  | neutrophilic granulocytes |
|  | neutrophil granulocytes |
|  | neutrophil leukocyte |
|  | neutrocytes |
|  | neutrophils |
|  | neutrophilic leukocytes |
|  | neutrophilic leukocyte |
|  | neutrophil |
|  | pmn granulocytes |
|  | polynuclear leukocyte |
|  | polymorphous leukocytes |
|  | pmn leukocyte |
|  | pmn granulocyte |
|  | polynuclear leukocytes |
|  | neutrophilic |

---

|  |  |
| --- | --- |
| NCIm ID | C0027950 |

---
